# Blood-based biomarkers GFAP/UCH-L1 for the diagnosis of mild traumatic brain injury (mTBI): a single-center implementation experience

**DOI:** 10.1007/s00068-026-03244-y

**Published:** 2026-06-24

**Authors:** Marc Maegele, Lucas Breidenbach, Janina Kaufmann, Yunus Keles, Eberhard Uhl, Peter Schroeer

**Affiliations:** 1 Department for Orthopedics, Trauma Surgery, and Sports Traumatology, Cologne-Merheim Medical Center (CMMC), Ostmerheimerstr. 200, D-51109 Cologne, Germany; 2 Institute for Research in Operative Medicine (IFOM), Witten/Herdecke University, Cologne-Merheim Campus, Ostmerheimerstr. 200, D-51109 Cologne, Germany; 3 Clinic for Trauma Surgery and Orthopedics, Hand, Spine Surgery and Sports Traumatology, Klinikum Oberberg GmbH, Gummersbach District Hospital, Wilhelm-Breckow-Allee 20, D-51643 Gummersbach, Germany; 4 Medical Care Center/Medizinisches Versorgungszentrum (MVZ) SYNLAB, Cologne-Merheim, Ostmerheimerstr. 200, D-51109 Cologne, Germany; 5 Department for Neurosurgery, University Hospital Gießen (UKGM), Klinikstr, 33, D-35392 Gießen, Germany; 6 Department for Orthopedics, Trauma Surgery, and Sports Traumatology, Institute for Research in Operative Medicine (IFOM), Witten/Herdecke University, Cologne-Merheim Campus, Merheim Medical Center (CMMC), Ostmerheimerstr. 200, D-51109, Cologne, Germany

**Keywords:** Traumatic brain injury (TBI), GCS, Cranial computed tomograpgy (brain CT scan), Biomarker, GFAP, UCH-L1

## Abstract

**Introduction:**

Mild traumatic brain injuries (mTBI) affect millions of people worldwide every year as one of the most common clinical presentations in the emergency department. Diagnosis is mainly based on clinical criteria and computed tomography scans. The use of computed tomography causes high costs, long waiting times in daily clinical practice and radiation exposure. GFAP (glial fibrillary acidic protein) and UCH-L1 (ubiquitin carboxyl-terminal hydrolase-L1) turned out to be potential biomarkers for the diagnosis of mTBI. This study retrospectively evaluates the possible use of these biomarkers combined as negative predictors for excluding brain injuries in patients with suspected mTBI in the emergency department.

**Methods:**

Adult patients (*n* = 320) registered in the emergency department at a level 1 trauma emergency center in Germany (Cologne Merheim Medical Center/CMMC) between 11/2023 and 04/2024, with suspected mTBI, Glasgow Coma Scale (GCS) score 13–15 and within 12 h after trauma were considered. All evaluable patients underwent cranial CT (cCT) scans and blood tests for GFAP and UCH-L1 serum concentrations.

**Results:**

Biomarkers GFAP and UCH-L1 were tested positive in 261 patients (82%) while CT detected intracranial injuries in only 29 patients (9%). Biomarkers combined had a sensitivity of 97% and a negative predictive value (NPV) of 98% in mTBI diagnosis with a negative CT scan.

**Conclusions:**

The biomarkers GFAP and UCH-L1 combined could play a potential clinical role in avoiding unnecessary cCT scans in emergency departments after mTBI, might reduce treatment times and reduce radiation exposure.

## Introduction

The number of patients with traumatic brain injuries (TBI) is steadily increasing, with approximately 50–60 million people worldwide suffering a TBI each year [1]. In high-income countries, falls among older people are the most common cause of TBI, and the prevalence is likely to continue to rise in the future due to demographic change [2]. Over 90% of cases treated in emergency departments are mild TBI (mTBI), defined as a total score of 13 to 15 on the Glasgow Coma Scale (GCS) [1]. The routine initial work-up for suspected TBI includes a careful and targeted medical history, followed by a neurological examination and computed tomography imaging of the head (cranial computed tomography (cCT)); the latter is ideal for visualizing different types of injuries and is crucial for identifying patients with TBI who require surgical treatment. In a retrospective analysis of the US National Hospital Ambulatory Medical Care database, which now includes nearly 5 million emergency department visits due to TBI, cCT was performed in 82% of cases [3], with traumatic changes detected in only 9% of the images and surgical intervention resulting in only 0.5% of these cases [3]. Accordingly, 94.5% of patients in this cohort were classified as having mTBI according to the GCS [3].

With the prevalence of TBI steadily increasing, the number of emergency cCT performed has risen dramatically over the past decade and a half [4]. According to a single-center observational study from the Netherlands, the total workload for radiologists in terms of relative value units (RVUs) to estimate workload — not only when on-call — quadrupled between 2006 and 2020 [4]. Computed tomography examinations, the number of which increased by more than 500% during the observation period, were mostly ordered in the context of head/polytrauma, suspected pulmonary embolism and aortic dissection [4]. The excessive use of CT imaging is also associated with increased radiation exposure, high costs, more complex procedures and longer stays in emergency departments. Against this background, there is growing interest in blood-based biomarkers for diagnostic and prognostic purposes, especially in patients with mTBI [1, 5].

**Fig. 1:**
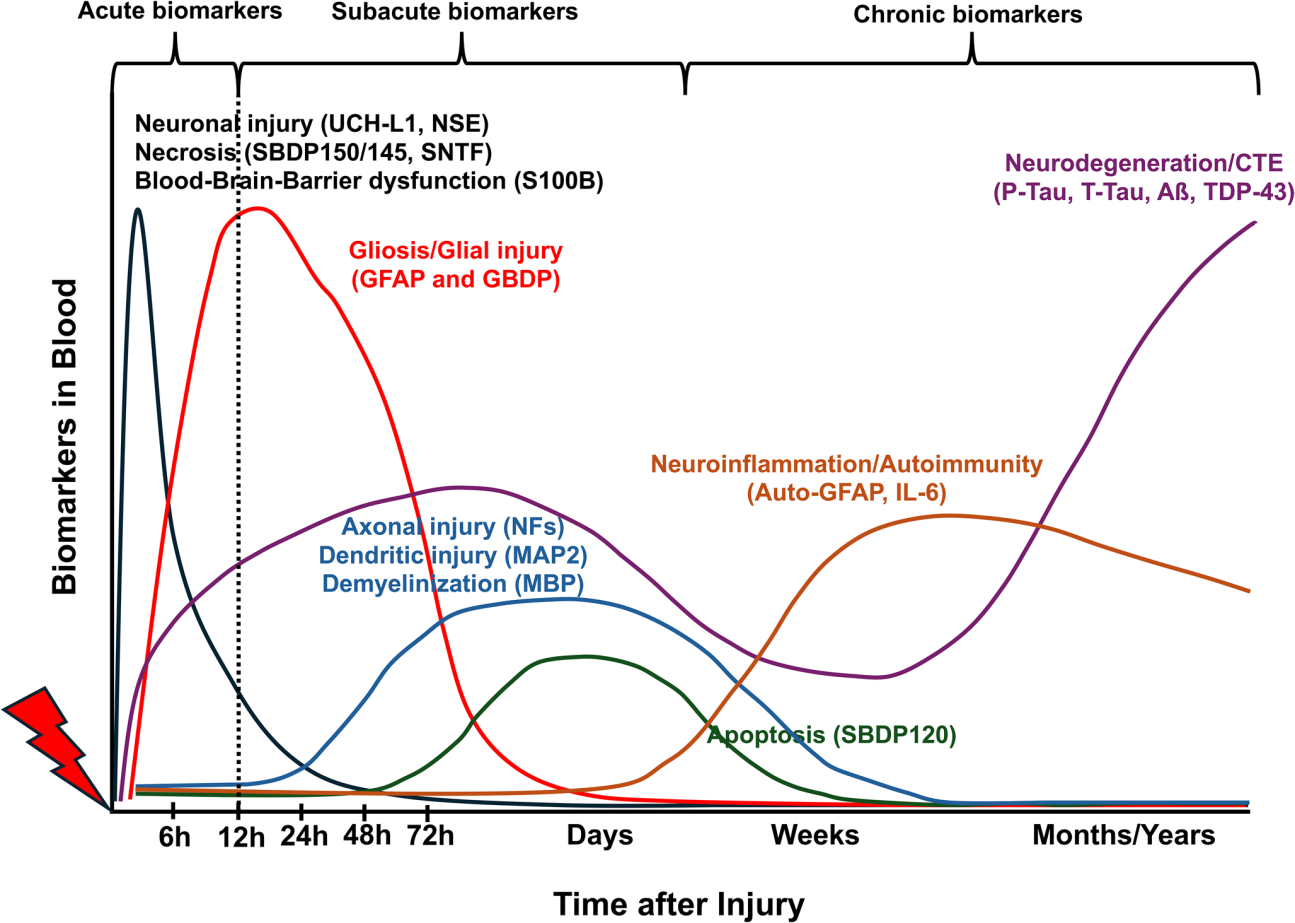
The continuum of blood-based protein biomarkers investigated to date for tracking different phases after traumatic brain injury (TBI). Acute biomarkers for detecting neuronal injury include UCH-L1 (ubiquitin C-terminal hydrolase-L1), NSE (neuron-specific enolase), necrosis markers SBDP150/145 (αII-spectrin cleavage product 150 kDa & 145 kDa), SNTF (αII-spectrin N-terminal fragment), S100B (glial calcium-binding protein S100B) and GFAP & BDP (glial fibrillary acidic protein & cleavage products). These biomarkers are detectable within 12 h of injury. Subacute biomarkers occur later up to weeks after injury and indicate delayed axonal injury, e.g. NF-H, M, L (neurofilament heavy, medium & light), demyelinization, e.g. MBP (myelin basic protein) and apoptosis, e.g. SBDP120 (αII-spectrin cleavage product 120 kDa). Chronic biomarkers reflecting neuroinflammation/autoimmunity and neurodegeneration are detectable weeks to months/years after injury and include autoimmune marker auto [GFAP] (autoantibody against GFAP), neurodegeneration markers Tau (tau protein), P-Tau (phosphorylated tau), Aβ (amyloid β-peptides) and TDP-43. (modified from [47])

Blood-based biomarkers with high sensitivity, sufficient specificity, and well-characterized release kinetics could be very helpful in the diagnosis and treatment of patients with mTBI, as they enable better stratification, optimization of diagnostics/workflows, and prognoses [5]. Unlike imaging, they also provide a more objective measure of brain damage, leading to a potentially more accurate classification of the injury sustained [5]. Even modern imaging is not sensitive enough in individual cases to accurately diagnose all forms and degrees of severity of TBI and predict its outcome [6, 7]. The most promising biomarkers currently available for acute diagnosis of suspected mTBI within the first 24 h are S100B, glial fibrillary acidic protein (GFAP), and ubiquitin C-terminal hydrolase L1 (UCH-L1) (Fig. 1); all three are now recommended for clinical use by various guidelines and consensus reports [8–10] and will be taken into account in the foreseeable future when revising the currently unsatisfactory TBI classification systems [11].

Based on the prospective ALERT-TBI study, a combined assay of GFAP and UCH-L1 was approved in the US in 2018 for supportive diagnostics and stratification with regard to cCT imaging in patients with mTBI [12], while the use of S100B in clinical observational studies has led to a reduction in cCT scans [5]. In a retrospective analysis, GFAP alone was also able to predict abnormalities in magnetic resonance imaging (MRI) that were not visible on the initial cCT scan [13]. The present study investigated the clinical utility of the blood-based and combined biomarkers GFAP and UCH-L1 against routine cCT diagnostics in the early diagnosis of patients with mTBI (GCS 13–15) in a level 1 trauma emergency center in Germany (Cologne-Merheim Medical Center/CMMC) and classified the results in the current scientific context.

## Materials and methods

### Glial fibrillary acidic protein (GFAP)

GFAP is an intermediate filament protein that occurs mainly in astrocytes and supports cell structure and stability [14]. When the brain is damaged, e.g. in TBI, stroke, tumor or neurodegenerative processes (Alzheimer disease), GFAP is released into the extracellular space and systemic circulation. It is detectable within one hour of injury and has a half-life of 24–48 h, depending on the extent of the injury and the test method used [15, 16]. An increase in GFAP concentration in blood or cerebrospinal fluid indicates damage or activation of astrocytes and a disruption of the blood-brain barrier [17].

### Ubiquitin C-terminal hydrolase L1 (UCH-L1)hydrolase L1 (UCH-L1)

UCH-L1 is an enzyme of the ubiquitin-proteasome system, an important mechanism for protein degradation in the cell by attaching or removing ubiquitin from metabolic proteins, abnormal proteins, or proteins damaged by oxidation [18]. Therefore, it is considered a signal for neuronal damage and cellular stress. UCH-L1 is systemically detectable within 30 min after injury, reaches its maximum concentration early after 8 h, and has a short half-life of 7–9 h [19]. The fact that UCH-L1 is produced and released by neurons with sequential kinetics makes it an attractive partner for GFAP in the context of early TBI diagnosis [5, 19–21]. Prospective observational data have now demonstrated superior sensitivity and specificity for the diagnosis of TBI when UCH-L1 and GFAP are combined on the day of injury, supporting the promising concept of combining blood-based biomarkers for assessment and prognosis, especially in mTBI [12, 22–24].

### Patients

Adult patients (> 18 years) with suspected mTBI (GCS 13–15) and clinical indication for cCT based on the assessment of the attending physician in the emergency department within 12 h of injury were enrolled in the study on a non-consecutive basis. The time window for patient enrollment was 6 months from 11/2023 to 04/2024. The study was designed as a control study to implement both biomarkers into routine clinical practice and the emergency department workflow. Blood samples for biomarker analysis were collected as part of routine emergency department assessment and used in accordance with good clinical practice; all demographic (age and gender), epidemiological (medical history), clinical (GCS), and cCT data, including time stamps, were retrieved from clinical records. The time intervals between injury and admission, biomarker/CT requirements, and result transmission for clinical decision-making were determined either directly, where possible, or indirectly from the medical documentation files.

### Biomarker evaluation

Peripheral blood samples were collected during initial contact in the emergency department and within 12 h of injury, and immediately forwarded to the central laboratory for biomarker evaluation. GFAP and UCH-L1 serum levels were determined using commercial chemiluminescent microparticle immunoassays (CMIA) on the Alinity i platform (Alinity i TBI test; ABBOTT Core Diagnostic, Abbott Park, IL 60064, USA) according to the manufacturer’s instructions [21]. The cut-off values for GFAP and UCH-L1 were 35 pg/mL and 400 pg/mL, respectively. For a positive test result, GFAP or UCH-L1 or both biomarkers together had to be above their respective cut-off values; conversely, for a negative test result, both biomarkers had to be below their respective cut-off values. The combined sensitivity and specificity of the GFAP and UCH-L1 biomarker test were determined to assess the overall performance of the test in predicting cCT positivity or negativity.

## Results

**Fig. 2:**
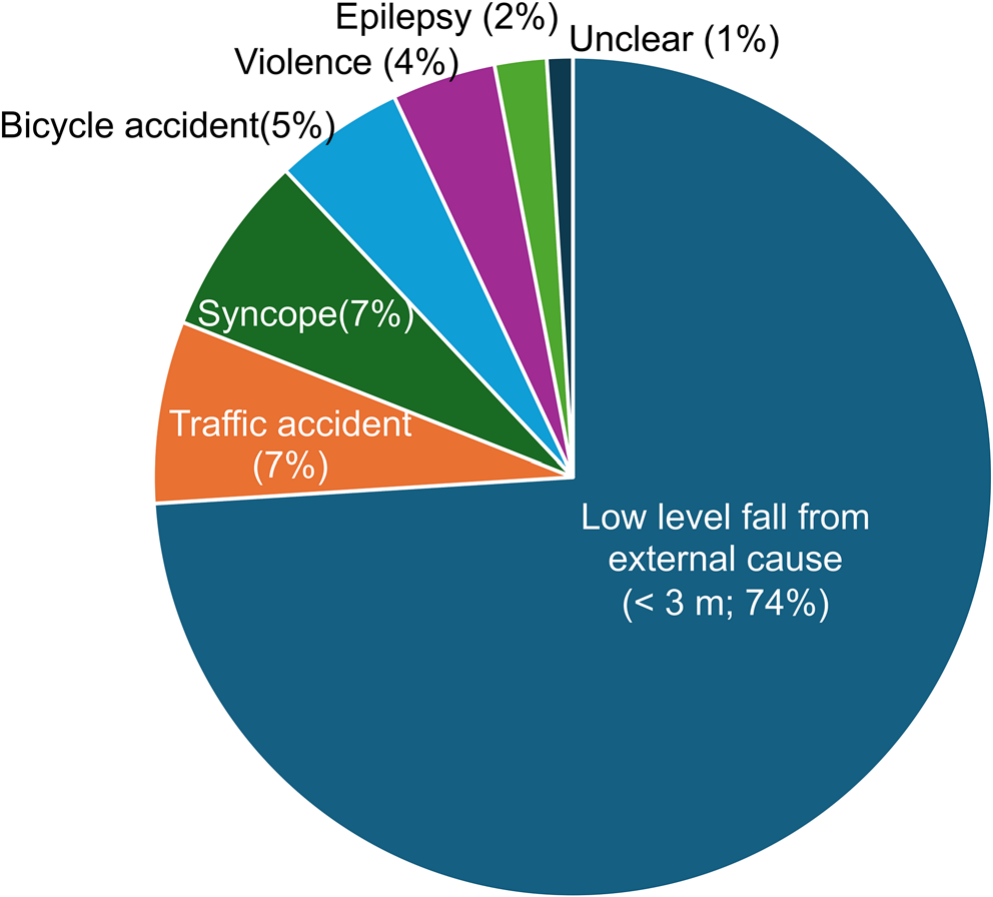
Causes of injury in the cases included

A total of 320 cases were included in the study on a non-consecutive basis during the observation period; 145 of these were men (45%) and 175 were women (55%). The median age was 79 years (IQR 61–87 years), with slightly above two-thirds (69%) of cases aged > 65 years. In 10 cases (3%), a GCS of 13 was recorded during the assessment in the emergency department, 41 (13%) cases were classified as GCS 14, and the remaining 269 (84%) as GCS 15. The Fig. 2 shows an overview of the causes of injury in the included cases. In all cases, a routine blood sample was taken as part of routine diagnostic work-up in the emergency department, from which the biomarkers were determined in addition to the routine diagnostics, and a cCT was performed. The electronic turn-around time for biomarker determination via the hospital information system was robust 17 min after blood collection and submission, and the time interval between admission to the emergency department and cCT without transmission of findings/report was 77 min on average (SD +/- 71 min). A positive blood alcohol level was recorded in 29/253 cases. As a result, the prevalence of a positive test result for the two combined biomarkers was 82% (261/320), while that for a positive cCT was 8% (25/320). In one case, the cCT showed abnormal findings while the biomarker test was negative; the cause of the cCT positivity in this case was a previous brain surgery with scarring. The Table 1 summarizes the underlying intracranial lesion/bleeding types of the cCT-positive cases. cCT positivity was primarily observed in patients with clinically impaired GCS 13 and 14 along with minor clinical symptoms but not in a consistent pattern.

**Table 1:** Overview of injury patterns in the included patients with positive cCT findings

Lesion/Bleeding type	Patients with positive cCT (n=25)
Subduralhematoma	10
Subarachnoid Hemorrhage	4
Subduralhematima + Subarachnoid Hemorrhage	4
Intraparenchymal Bleeding + Subduralhematoma	2
Intraparenchymal Bleeding + Subarachnoidal hemorrhage	2
Epiduralhematoma	1
Epiduralhematoma + Subarachnoid Hemorrhage	1
Intraparenchymal Bleeding	1

**Fig. 3:**
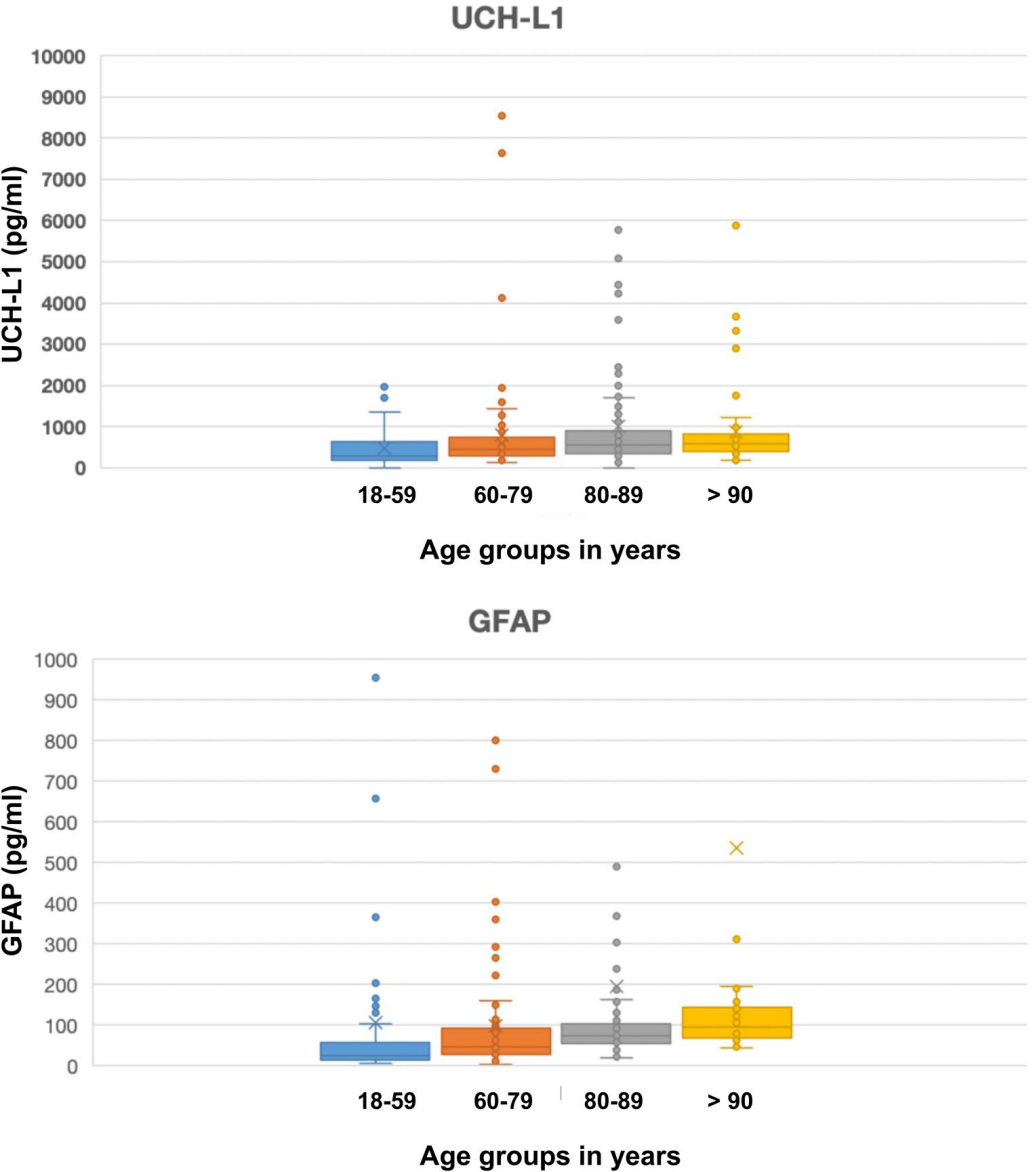
Step-wise increase in measured biomarker levels for UCH-L1 (upper panel) and GFAP (lower panel) according to age groups. The increase was more pronounced for levels of GFAP

The median biomarker levels for GFAP across all patients were 63 pg/ml (IQR 30–104), for biomarker-positive patients 82 pg/ml (IQR 58–121), and for biomarker-negative patients 22 pg/ml (IQR 15–27); for UCH-L1, the median levels were 508 pg/ml (IQR 288–777), 716 pg/ml (IQR 561–1220), and 268 pg/ml (IQR 197–332), respectively. The diagnostic sensitivity of the combined biomarker test across all cases with mTBI defined by GCS 13–15 was 97% (95% CI 93.8–98.1) with a specificity of 20% (95% CI 15.6–24.3) and an NPV of 98% (95% CI 96.8–99.7). The average age of the biomarker-positive population was significantly higher compared to the biomarker-negative population (77 ± 16 years versus 53 ± 19 years) with a slightly reduced but non-significant average GCS. To further investigate this finding, the population was stratified into age groups based on their GFAP and UCH-L1 expression (Fig. 3). While no significant age differences were observed for UCH-L1, GFAP showed a significant increase in older age groups (*p* < 0.01), with the majority of the population well above the positivity threshold from the age of 80 years onwards.

## Discussion

Over the last two decades, biomarkers have evolved from research tools to clinical applications [11]. In the present study, the combined and blood-based biomarkers GFAP and UCH-L1 excluded cCT-visible lesions in mTBI (GCS 13–15) within 12 h of injury with a sensitivity of 97% and a negative predictive value (NPV) of 98%. This result confirms earlier reports suggesting superior sensitivity and specificity with combined biomarker or biomarker panel use compared to single protein analysis [19, 25]. In the prospective ALERT-TBI multicenter study, a blood test using GFAP and UCH-L1, which would identify cCT-positive findings, had higher sensitivity (0.98) and specificity (0.36 [12]) than protein S100B in the validation studies for the Scandinavian TBI guideline (sensitivity 0.94 and 0.19 [26, 27]). The NPV in the ALERT-TBI study, which included almost 2,000 patients, for ruling out a traumatic lesion visible on cCT was 0.996 (95% CI 0.987–0.999 [12]). In two single-center observational studies from Italy involving in total over 200 cases of mTBI (GCS 13–15), an NPV of 100% was even observed [28, 29]; in a Dutch multicenter observational study involving over 250 cases, the NPV was just below this value [30]. In the prospective BRIANI study, which included 1,500 mTBI cases from 16 emergency departments in Spain and France between 2019 and 2021, the sensitivity of the combined GFAP/UCH-L1 blood test was calculated at 98% with an NPV of 99% (95% CI 97.1–99.8 [31]). When the patient with a false-negative test result due to prior brain surgery was excluded from the present analysis, the NPV for the cohort reported here was also 100% with 100% sensitivity.

As in most other studies, the combined specificity of both biomarkers for a positive cCT finding was also low in the present study at only 20%, which can be explained by the high number of elderly patients in the analyzed cohort: within the biomarker-positive group (*n* = 261; 82%) the average age was 77 years. Increasing age and age-related neurodegenerative diseases (e.g., Alzheimer’s dementia or other dementias) are associated per se with higher baseline levels of TBI biomarkers, including GFAP and UCH-L1 [32–36]. This leads to a reduced concentration range in which acute brain injuries can be differentiated, and the introduction of age- and/or comorbidity-specific cut-off values seems reasonable in order to reduce unnecessary cCT examinations in older adults as well [33]. In pediatrics, there remains a lack of clear data on the usability of GFAP, UCH-L1, or S100B measurements in cases of suspected TBI, although there is growing evidence that blood-based biomarkers are also associated with intracranial injuries in children. In children, the current clinical decision rules, e.g., PECARN, still offer the best guidance for the use of imaging techniques following the ALARA principle (As Low As Reasonably Achievable [37–39]).

At present, protein S100B remains the most thoroughly researched biomarker in the context of TBI and has been an integral part of the Scandinavian guidelines for the treatment of minimal, mild, and moderate head injuries in adults since 2013, followed shortly thereafter by French guidelines [8–10]. According to these guidelines, in cases of isolated mild head injuries, low risk of intracranial hemorrhage and concentrations < 0.10 mcg/l, a cCT can be omitted and the patient be discharged with a patient information sheet [8]. Similar to GFAP, S100B is released into the bloodstream after damage to glial cells. It has excellent diagnostic sensitivity for identifying patients who are likely to have a positive cCT result. S100B has been shown to be cost-effective and safe for reducing the number of unnecessary cCT examinations [26, 27, 40]. However, these advantages must be weighed against the overall low specificity of S100B for the brain, which is associated with a relatively high number of negative cCT examinations. In addition, due to its short half-life, the indication for S100B is limited to diagnosis within 6 h of injury [41]. In the present study, the average time window between the estimated time of injury and presentation to the emergency department was over 7 h; the delayed presentation most likely reflects clinical reality in patients with mTBI. Due to their delayed detectability, UCH-L1 and GFAP allow for a larger time window in the acute phase and are approved for diagnosis up to 12 h after injury. In a direct comparison with GFAP, S100B was significantly inferior to GFAP in predicting CT lesions within 4 h of injury in predominantly mTBI, with an AUC of 0.78 (0.67–0.89) versus 0.84 (0.73–0.95) for GFAP [42].

### Two recent meta-analyses

In a recent meta-analysis, the combined measurement of GFAP and UCH-L1 enabled the exclusion of intracranial injury after mTBI in adults with a sensitivity and an NPV of 100% each [43]. Theoretically, this study showed that the routine use of diagnostic cCT scans could have been reduced by 31% by the use of the two biomarkers. A second recent meta-analysis including 13 studies essentially confirmed these results [44]. Further studies underscore the strong performance of GFAP in particular for lesions detected by cCT [11]; with an AUC of 0.78 (95% CI 0.73–0.83), GFAP even detected lesions exclusively in follow-up MRI in initially CT-negative patients within 7–18 days after injury [13]. The diagnostic added value of UCH-L1 compared to GFAP alone is under discussion. However, since UCH-L1 is detectable earlier than GFAP and reaches its peak value also earlier [19], it is likely that UCH-L1 is necessary to close the early diagnostic gap of GFAP.

### Potential savings for cCT examinations and inpatient monitoring using biomarkers

**Fig. 4:**
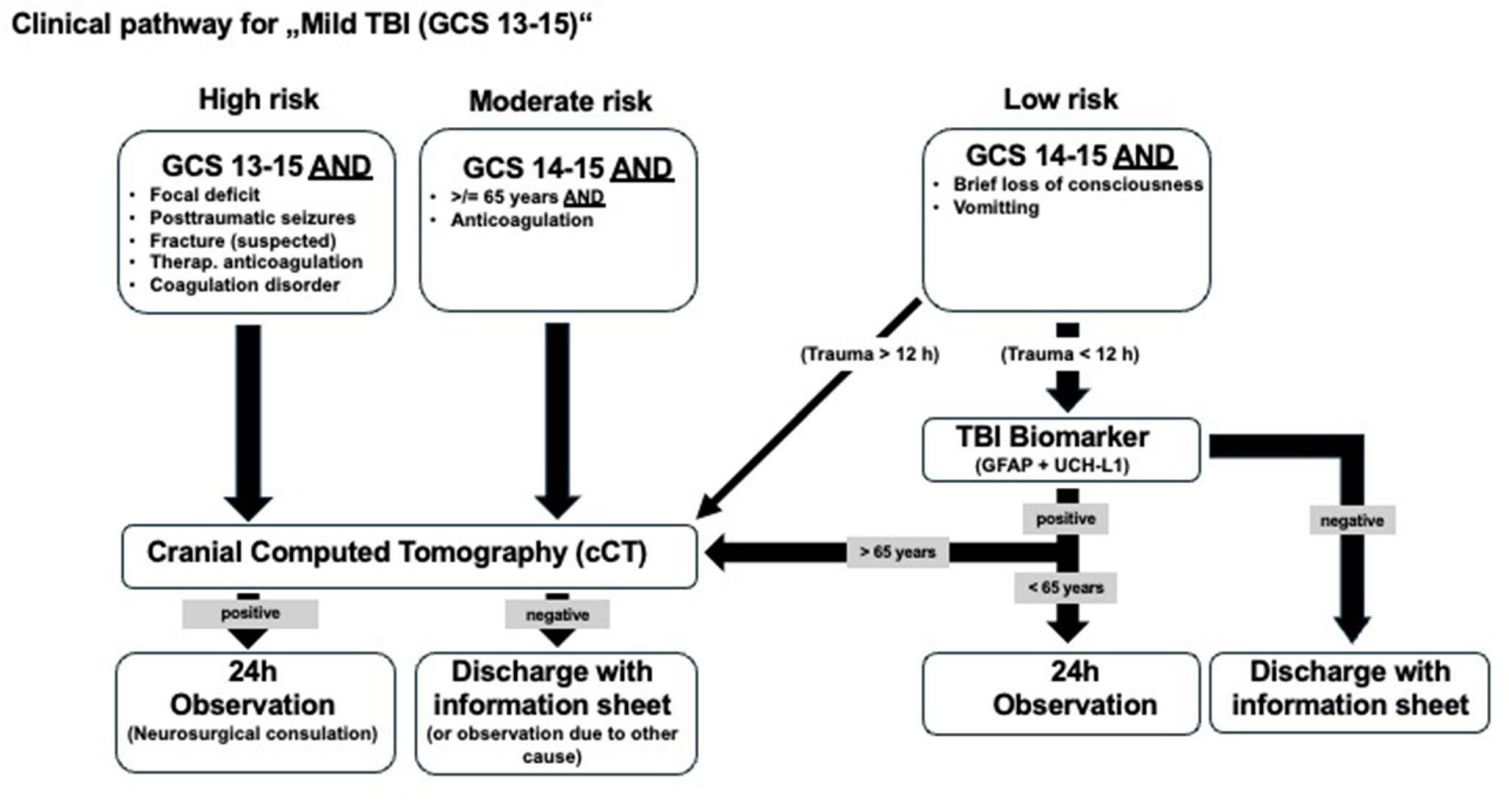
Clinical pathway - initially developed and adopted for S100B - modified to UCH-L1 and GFAP for the diagnosis of patients with mild TBI (GCS 13–15; modified from (8 and 40)). Patients with mild TBI (GCS 14–15) at low risk presenting within 12 h of injury are subjected to biomarker GFAP and UCH-L1 assessment upon ED arrival. In case of a negative result, these patients can be discharged with a patient information sheet. In case of a postive result and if < 65 years of age patients are being admitted for 24 h observation; if > 65 years of age, patients undergo cCT diagnostics. Patients with GCS 13–15 at moderate to high risk undergo routine cCT diagnostics

The results and clinical courses of 163 cases (GCS 14–15) in the cohort reported here were reviewed using the algorithm adapted from S100B to GFAP/UCH-L1 in the Scandinavian guidelines for the management of mTBI with regard to possible reductions in cCT scans and inpatient monitoring (Fig. 4). In 40% of cases (62/163), the use of the combined GFAP/UCH-L1 biomarker would have made it possible to dispense with a cCT in the emergency department; in every third case (42 versus 66), it would have made it possible to dispense with inpatient admission for monitoring. The cCT saving effects would have primarily benefited patients at low risk with a negative biomarker test (*n* = 29) that could have been discharged right away and those with a positive biomarker patients but < 65 years that would have been admitted but without a cCT performed (*n* = 33). The percentage reduction in cCT scans and thus reduced exposure to potentially harmful radiation in the emergency department reported here corresponds to the results of the recently published meta-analysis [43]. Ionizing radiation is one of the very few agreed risk factors for central nervous system (CNS) tumors and the only modifiable factor but information on quantitative estimates of radiogenic risk for different types of CNS tumors is still scarce [45]. The EPI-CT cohort study on over 650,000 patients under 22 years of age reported a dose-response relationship between CT-related radiation exposure and brain cancer [46] but the risk is likely to become less relevant with increasing age.

### Integration into German guidelines for TBI

Due to increasing evidence, the German AWMF-S3 guideline “Traumatic Brain Injury in Adults,” which is currently being revised, has now also included the use of blood-based biomarkers in its recommendations for the diagnosis of mTBI with a GCS 13–15. In addition to the accepted and mandatory indications for performing a cCT examination in cases of TBI, the biomarkers discussed here are recommended for the first time in cases of optional cCT indication and low risk of intracranial injury. For all three biomarkers, the cut-offs described for predicting a positive cCT finding are still method- and/or laboratory-specific, which necessitates the establishment of certified, standardized, and thus reliable reference materials and methods. For clinical decision-making, the reference limits and cut-offs recommended by the various manufacturers must therefore be taken into account in conjunction with laboratory-specific data and clinical presentation, which are summarized in Table Table 2. Alternative applications for the blood-based biomarkers described here could in the future extend to prognostic assessment and treatment monitoring in cases of severe TBI, as well as, where appropriate, to patient stratification into specific treatment and rehabilitation programs.

**Table 2:** Diagnostic performance of FDA- or CE-approved assays for GFAP, ubiquitin C-terminal hydrolase L1 (UCH-L1), and S100B for predicting traumatic intracranial injuries in cCT during the acute phase after injury

Assay/Test	Reference ranges^f^	Time after injury (h)	Cutofff	TBI (GCS)	Sensitivity(95% CI)	Specificity(95% CI)	Negative Predictive Value (NPV)(95% KI)	Postive predictive Value (PPV)(95% KI)	Reference
**GFAP+UCH-L1**									
Banyan BTI[Serum]	G: 10–320U: 80–2560	0–12	G: 22U: 327	9–1514–15	0.976 (0.931–0.995) 0.973 (0.924–0.994)	0.364 (0.342–0.387) 0.367 (0.345–0.390)	0.996 (0.996–0.999) 0.995 (0.987–0.999)	0.092 (0.079–0.112) 0.088 (0.073–0.105)	Bazarian(2018)
i-STAT Alinity TBI Plasma Test^a^[EDTA Plasma]	G: 30–10000U: 200–3200	0–12 0–12 0–2	G: 30U: 360G: 30U: 360G: 30U: 360	13–15 15 15	0.958 (0.906–0.982) 0.957 (0.896–0.983) 1.00 (0.723–1.00)	0.404 (0.382–0.427) 0.411 (0.387–0.434) 0.356 (0.304–0.412)	0.993 (0.985–0.997) 0.994 (0.985–0.998) 1.00 (0.965–1.00)	0.098 (0.082–0.116) 0.083 (0.068–0.098) 0.050 (0.027–0.089)	Bazarian(2021)US FDA(2024)
i-STAT TBI^a^[EDTA Vollblut]	G: 47–10000U: 87–3200	0–24	G: 65U: 360		0.965	0.403	0.965 (adj: 0.994)	0.40 (adj: 0.094)	US FDA(2024)
Alinity i/ARCH TBI^a^[Plasma + Serum]	G: 6–42000U: 26–25000	0–12	G: 35U: 400	9–15	0.967 (0.917–0.987)	0.401 (0.378–0.424)	0.994 (0.986–0.998)	0.098 (0.082–0.116)	US FDA(2023)
VIDAS^®^ TBI^b^ (GFAP+UCH-L1)[Serum]	G: 10–320U: 80–2560	0–12	G: 22U: 327	13–15	0.967 (0.917–0.991)	0.412 (0.389–0.435)	0.995 (0.986–0.999)	0.099 (0.083–0.118)	US FDA(2024)
**S100B**									
Elecsys^®^S100^c^ [Serum]	S: 0.005–39^d^ S: 0.015–30^e^	0–3	S: 0.105	13–15	0.988 (0.965–1.00)	0.329 (0.030–0.359)	0.997 (0.991–1.00)	0.11 (0.088–0.133)	Roche(2023)

^a^Abbott; ^b^bioMerieux.; ^c^Roche; ^d^Cobas e411, e601/e602; ^e^Cobas e402, Cobas e801; ^f^G: GFAP (pg/mL), U: UCH-L1 (pg/mL), S: S100B (mg/L)BTI: Brain Trauma Indicator; GCS: Glasgow Coma Scale; GFAP: Glial Fibrillary Acidic Protein; KI: Konfidenzintervall; S100B: S100B Calciumbindendes Protein; TBI: Traumatic Brain Injury; UCH-L1: Ubiquitin C-terminal

## Data Availability

No datasets were generated or analysed during the current study.
